# Exploring the core symptoms and influencing factors of fatigue-related symptom clusters in patients with HIV/AIDS

**DOI:** 10.3389/fpubh.2026.1727263

**Published:** 2026-03-03

**Authors:** Dan Xu, Jianghui Zhang, Lan Shen, Haowen Chen, Jia Li

**Affiliations:** 1 Department of Nursing, Zhuhai Campus of Zunyi Medical University, Zhuhai, China; 2 Department of Infectious Diseases, The Affiliated Hospital of Guizhou Medical University, Guiyang, China; 3 Department of Infectious Diseases, The Fifth Affiliated Hospital of Zunyi Medical University, Zhuhai, China; 4 Department of Nursing, Macau Special Administrative Region, Kiang Wu Nursing College of Macau, Macau, Macao SAR, China

**Keywords:** fatigue, HIV/AIDS, network analysis, symptom cluster, symptom management

## Abstract

**Objective:**

To explore the core symptoms and associated factors of fatigue-related symptom clusters in people living with HIV/AIDS during follow-up, with the aim of informing precision-based symptom management strategies.

**Methods:**

Fatigue-related symptom clusters in patients with HIV/AIDS were assessed using the Fatigue Self-Assessment Scale, the Pittsburgh Sleep Quality Index, the Sexual Dysfunction Scale, and the Groningen Frailty Indicator. Symptom networks were constructed using R software to identify central symptoms based on network metrics. Univariate analysis and multivariate linear regression were then performed to explore potential factors associated with these core symptoms.

**Results:**

Among male patients living with HIV/AIDS, the three most prevalent symptoms within the fatigue-related symptom cluster were fatigue (86.32%), sleep disturbances (44.63%), and weakness (43.32%). In female patients, the top three symptoms were fatigue (73.04%), sexual dysfunction (72.17%), and sleep disturbances (45.22%). In the symptom network for males, the three nodes with the highest strength centrality were sleep disturbances (D5, rs = 1.61), fatigue severity and type (C1, rs = 1.60), and sleep quality (D1, rs = 1.52). In females, the top three most central symptoms were difficulty achieving orgasm (B4, rs = 2.16), daytime functioning (D7, rs = 1.66), and sexual satisfaction (B5, rs = 1.09). Partner’s HIV status, employment status, number of sexual partners, and duration of ART were identified as significant factors influencing the core symptoms within the fatigue-related symptom cluster (*p* < 0.05).

**Conclusion:**

In the fatigue-related symptom clusters among people living with HIV/AIDS, the core symptoms shared by both male and female patients namely sleep disturbances and impaired daytime functioning indicate that sleep problems are a central feature of these clusters. Accordingly, sleep issues may be considered a primary target for symptom management in this population. Healthcare professionals should develop targeted interventions based on these core symptoms and their influencing factors to improve the efficiency and precision of symptom management for individuals living with HIV/AIDS.

## Introduction

With the widespread implementation and promotion of antiretroviral therapy (ART), HIV infection has shifted from a fatal disease to a manageable chronic condition. The life expectancy of people living with HIV/AIDS is now approaching that of the general, uninfected population. As a result, efforts in HIV/AIDS prevention and control have increasingly focused on reducing the number of new infections and improving the quality of life for those living with the disease (1). In response, the Joint United Nations Programme on HIV/AIDS (UNAIDS) proposed a “fourth 90” target in 2019, building on the original “90–90–90” goals. This additional target aims to ensure that 90% of individuals with viral suppression also achieve a good quality of life (2). However, due to the combined effects of the disease itself, antiretroviral medications, and psychosocial factors, The people living with HIV/AIDS (PLWHA) often experience multiple concurrent symptoms during treatment. These symptoms interact with and influence one another, forming symptom clusters. Compared to isolated symptoms, the synergistic effects within symptom clusters can significantly increase symptom burden. If left unmanaged, they may accelerate the decline in patients’ functional status and quality of life (3).

Studies have identified fatigue, sleep disturbances, weakness, and sexual dysfunction as some of the most distressing and severe symptoms experienced by people living with HIV/AIDS (4, 5). These symptoms often co-occur and collectively form a fatigue-related symptom cluster (4, 6, 7). This cluster has been shown to negatively impact treatment adherence, therapeutic outcomes, and overall quality of life, ultimately contributing to increased mortality in this population (8–11). However, due to the large number of interrelated and interacting symptoms within the cluster, effective intervention remains challenging. The absence of clearly defined therapeutic targets has limited the precision and efficiency of symptom management strategies, thereby undermining the potential advantages of symptom cluster–based interventions.

Network analysis is an analytical approach that explores the complex interrelationships among symptoms by examining attributes such as centrality, accuracy, and stability within a network structure. It enables the identification of core symptoms, bridge symptoms, and other key features (12). By reflecting the interaction mechanisms of symptoms in real-world settings, network analysis provides precise intervention targets and optimizes symptom management strategies, thereby enhancing the efficiency and accuracy of clinical interventions (13). Accordingly, this study investigates the current status of fatigue-related symptom clusters among people living with HIV/AIDS and employs network analysis to identify core symptoms and their influencing factors. The ultimate goal is to provide a foundation for developing personalized and targeted symptom management strategies.

## Materials and methods

### Ethics approval

This study was approved (or granted exemption) by the ethics committee of The Fifth Affiliated Hospital of Zunyi Medical University (approval no. [2023]2023KY014). We certify that the study was performed in accordance with the ethical standards as laid down in the 1964 Declaration of Helsinki and its later amendments.

### Source of study subjects and sampling methods

**Figure 1:**
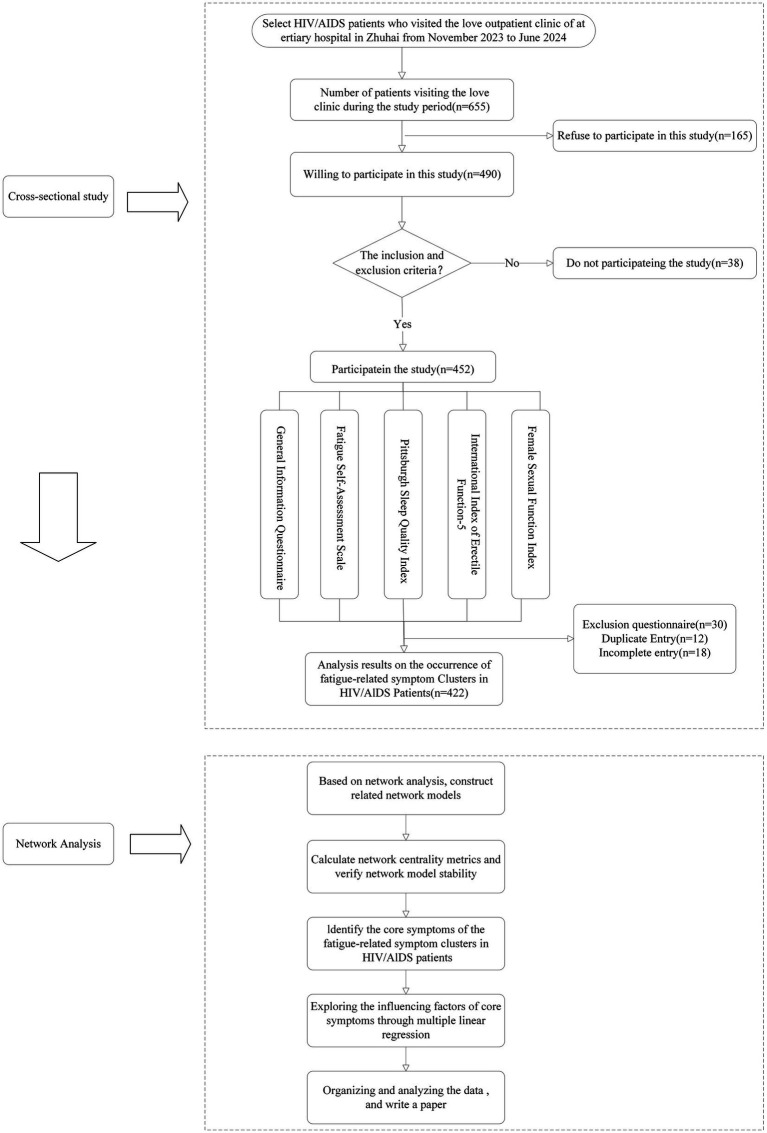
Then study design flow chart.

Using convenience sampling HIV/AlDS patients who visited the Love Clinic of a tertiary hospital in Zhuhai from November 2023 to June 2024were selected. The study design flow chart is provided in Figure 1.

### Study participants

This study employed a convenience sampling method to conduct a cross-sectional survey of HIV/AIDS patients attending the Love Clinic at a tertiary general hospital in Zhuhai, China, between November 2023 and June 2024. Inclusion criteria: (1) Diagnosed as HIV-positive; (2) Aged ≥18 years; (3) Clear consciousness and the ability to communicate and read normally; (4) Signed informed consent and voluntarily participated in the study. Exclusion criteria: (1) Presence of severe HIV-related neurocognitive disorders or other major physical illnesses; (2) History of chronic conditions such as diabetes, hypertension, or coronary heart disease; (3) Participation in other HIV/AIDS-related research studies.

### Sample size calculation

Currently, there is no consensus on the sample size calculation method for network analysis, and this remains an area for future research. The prevailing approach is to assess the reliability of network analysis results through post-hoc stability testing (14). Using the Kendall rough sampling principle (15) (which suggests that the sample size should be 5–10 times the number of predictor variables), we estimate the minimum sample size for this study, which involves 23 variables from the scales, to be between 115 and 230 participants. Considering a 20% attrition rate, the final sample size was determined to be 276 participants. Stability testing was then conducted to assess the reliability of the network analysis results. This study was approved by the Ethics Committee of the hospital where the research was conducted ([2023]2023KY014).

### Survey instruments

#### General information questionnaire

A self-designed questionnaire was used to collect general demographic, socio-economic, and disease-related data from HIV/AIDS patients. Demographic data: Age, gender, number of children, household composition, marital status, height, weight, sexual orientation, ethnicity, and HIV status of sexual partners. Socio-economic data: Income level, education level, marital status, employment status, and residence. Disease-related data: ART status, duration of ART treatment, presence of comorbidities, CD4 + T lymphocyte count, duration of HIV infection, and mode of transmission. Disease-related data were extracted by the investigator from follow-up records.

#### Fatigue self-assessment scale

The Fatigue Self-Assessment Scale (FSAS) consists of two dimensions: fatigue type and severity, and fatigue characteristics, with a total of 23 items (16). Items 1–22 are rated on a 5-point scale, ranging from 0 to 4 points. The standard score for fatigue type and severity ranges from 0 to 100, with higher scores indicating more severe fatigue. The severity levels are categorized into four levels: negligible, mild, moderate, and severe. The fatigue characteristics score also ranges from 0 to 100, with higher scores indicating more prominent fatigue characteristics. The severity levels are categorized into five levels: negligible, slight, moderate, marked, and very marked. FSAS has been validated and shown to possess good reliability and validity (17), and it can be used to assess fatigue type, severity, and characteristics in individuals aged 18 years and older across various disease populations.

#### Pittsburgh sleep quality index

The Pittsburgh Sleep Quality Index (PSQI) is primarily used to assess the sleep quality of participants over the past month. It consists of seven dimensions: sleep quality, sleep latency, sleep duration, sleep efficiency, sleep disturbances, use of sleep medications, and daytime dysfunction, with a total of 19 self-reported items and 5 items rated by others (18). The 19th self-reported item and the 5 items rated by others are not included in the scoring. The total score ranges from 0 to 21, with a score greater than 5 indicating poor sleep quality. PSQI has been validated and demonstrated to have good internal consistency and reliability (19).

#### International index of erectile Function-5

The International Index of Erectile Function-5 is a tool used to assess erectile function, consisting of three dimensions: erectile confidence, erectile function, and sexual satisfaction (20). The scale includes 5 items, each rated from 0 to 5 points. The total score ranges from 0 to 25, with scores less than 7 indicating severe erectile dysfunction, scores between 8 and 11 indicating moderate erectile dysfunction, scores between 12 and 21 indicating mild erectile dysfunction, and scores greater than 21 indicating normal erectile function (21).

### Female sexual function index

The Female Sexual Function Index (FSFI) is a scale used to assess female sexual function, consisting of six dimensions: sexual desire, sexual arousal, vaginal lubrication, orgasm, sexual satisfaction, and pain during intercourse. It includes a total of 19 items, with scores ranging from 0 to 5 or 1 to 5 for each item. The total score can range up to 36 points, with a score below 23.45 indicating the presence of sexual dysfunction (22). Chinese scholar Sun Xiaoguang translated and validated the scale, confirming its good reliability and validity (23).

### Groningen frailty Indicator

The Groningen Frailty Indicator (GFI) consists of four dimensions: physical, cognitive, social, and psychological, with a total of 15 items. Each item is scored on a 0–1 scale, and a score of ≥4 points indicates frailty (24). In 2022, Huang Yun zhi and colleagues translated and adapted the GFI into a Chinese version, which was validated to have good reliability and validity. This scale is suitable for use as a self-assessment tool in frailty evaluation studies within populations (25).

#### Data collection

Data were collected using paper-based questionnaires, administered by two trained nursing researchers. These researchers explained the purpose and significance of the study to the participants using standardized instructions. After obtaining informed consent, participants were asked to complete the questionnaire based on their personal circumstances. The questionnaires were to be filled out on-site. For illiterate participants, the investigators assisted by reading the questions and recording the answers through a question-and-answer format. Once completed, the questionnaires were immediately collected and checked. Any incomplete or unclear responses were addressed by communicating with the participants on-site.

### Statistical methods

#### Descriptive analysis

Data were entered and analyzed using EpiData 3.1 software with double-entry by two researchers. The occurrence of symptoms and the associated influencing factors of core symptoms in the fatigue-related symptom cluster of HIV/AIDS patients were analyzed. Normally distributed continuous data are presented as mean ± standard deviation (X̅ ± S), with comparisons between two groups made using the *t*-test and comparisons among multiple groups made using analysis of variance (ANOVA). Non-normally distributed data are presented as median and interquartile range. Categorical data are presented as frequency and percentage. Demographic, socio-economic, and disease-related data were analyzed for their impact on core symptoms using chi-square tests and one-way ANOVA, with a *p* < 0.05 considered statistically significant. Multivariate linear regression analysis was used to examine the impact of demographic, socio-economic, and disease-related factors on core symptoms, with a *p* < 0.05 considered statistically significant.

#### Network analysis

R software (version 4.3.0) was used for network analysis of the cross-sectional data. Using the EBICglasso function (26), symptom network structures for fatigue-related symptom clusters in male and female HIV/AIDS patients were constructed based on Spearman’s rank correlation. Each dimension of the scales served as a node, with edges representing the correlations between nodes. The thickness of the edges indicates the strength of the correlation, with thicker edges representing stronger correlations. The correlation between edges is referred to as edge weight (27). The CentralityPlot function was used to generate centrality measures, including strength (rs), closeness centrality (rc), and betweenness centrality (rb), to evaluate the importance, proximity, and potential of symptoms as bridging symptoms within the network. Strength reflects the importance of a node in the network, calculated as the sum of the absolute edge weights between the node and all other nodes. Nodes with greater strength contribute more significantly to the network structure and play a role in promoting the overall development of the network. Closeness centrality measures how close a node is to all other nodes, calculated by the inverse of the sum of distances between nodes. A higher value indicates that the symptom is closer to others and more likely to be at the center of the network. Betweenness centrality assesses the potential for a node to act as a bridge within the network, measured by counting the number of shortest paths between any two nodes that pass through the node. A higher value suggests that the symptom is more likely to be a bridging symptom (28). Bootstrapping analysis was performed using the Corstability function, with 1,000 non-parametric resampling tests to assess network stability. A stability coefficient greater than 0.25 indicates an acceptable network model stability, and a coefficient greater than or equal to 0.5 indicates good stability (14).

## Results

A total of 452 questionnaires were distributed in this study. Among them, 12 were duplicates and 18 were incomplete. After excluding these, 422 valid questionnaires remained, yielding a response rate of 93.36%. A total of 422 patients were included, with an average age of 43.28 ± 12.24 years. Further details can be found in Tables 1–3.

**Table 1:** Demographic characteristics of the patients (*n* = 422).

Item	Category	Frequency	Percentage (%)
Gender	Male	307	72.75
	Female	115	27.25
Ethnicity	Han	383	90.76
	Minorities	39	9.24
Marital status	Single	209	49.53
	Married	164	38.86
	Otherwise	49	11.61
Partner’s HIV status	Yes	97	22.99
	No	325	77.01
Sexual orientation	Heterosexual	271	64.22
	Homosexual	117	27.72
	Bisexual	34	8.06
Number of sexual partners	0	33	7.82
	1	309	73.22
	≥2	80	18.96
Number of children	0	172	40.76
	1	107	25.35
	≥2	143	33.89
Living arrangement	Living alone	193	39.96
	Spouse	126	26.09
	Children	86	17.81
	Parents	43	8.91
	Otherwise	35	7.25
BMI	Underweight	46	10.90
	Normal weight	265	62.80
	Overweight	94	22.27
	Obesity	17	4.03

**Table 2:** Socioeconomic characteristics of patients (*n* = 422).

Item	Category	Frequency	Percentage (%)
Education level	No formal education	9	2.13
	Lower secondary education or below	240	56.87
	Lower secondary education or above	173	41.00
Place of residence	Owner-occupied housing	176	41.71
	Rented housing	202	47.87
	Other	44	10.42
Economic income	<1,000 RMB	86	20.38
	1,000–3,000 RMB	70	16.59
	3,000–5,000 RMB	144	34.12
	≥5,000 RMB	122	28.91
Employment status	Unemployed	81	19.19
	Temporary or short-term employment	69	16.35
	Permanent or long-term stable employment	239	56.64
	Agricultural employment	14	3.32
	Retired	19	4.50
Health insurance coverage	Local health insurance	281	66.59
	No-Local health insurance	100	23.70
	Other insurance	9	2.13
	Uninsured	32	7.58

Multiple responses allowed; BMI, Body Mass Index.

**Table 3:** Clinical characteristics of the participants (*n* = 422).

Item	Category	Frequency	Percentage (%)
Comorbidities	Yes	28	6.64
	No	394	93.36
CD4 + T-cell count	<50	12	2.84
	50–350	106	25.13
	350–500	117	27.72
	≥500	187	44.31
Years since HIV infection	<1 year	47	11.14
	1–5 years	183	43.36
	5–10 years	85	20.14
	≥10 years	107	25.36
Duration of ART	<1 year	47	11.14
	1–3 years	102	24.17
	3–5 years	95	22.51
	≥5 years	178	42.18
Route of transmission	Heterosexual intercourse	234	55.45
	Homosexual intercourse	143	33.89
	Intravenous drug use	25	5.92
	Blood transfusion	2	0.47
	Unknown	18	4.27

### Presence of comorbidities, recent CD4 count, duration since HIV infection, duration of ART, and route of transmission

#### Prevalence of symptoms in the fatigue-related symptom cluster in HIV/AIDS patients

Among male HIV/AIDS patients, the three most prevalent symptoms in the fatigue-related symptom cluster were fatigue (86.32%), sleep problems (44.63%), and frailty (43.32%). For female HIV/AIDS patients, the most prevalent symptoms were fatigue (73.04%), sexual dysfunction (72.17%), and sleep problems (45.22%). Detailed information can be found in Table 4.

**Table 4:** Prevalence and symptom scores of the fatigue-related symptom cluster among patients with HIV/AIDS (*n* = 422).

Item	Prevalence [*n* (%)]	Score ( $\bar{X}\pm S$ )
Fatigue	265^m^(86.32)	20.20 ± 18.14
	84^w^(73.04)	19.39 ± 17.61
Sleep disturbances	137^m^(44.63)	5.65 ± 3.09
	52^w^(45.22)	5.65 ± 3.27
Frailty	133^m^(43.32)	3.25 ± 2.11
	51^w^(44.35)	3.36 ± 2.19
Sexual dysfunction	110^m^(35.83)	21.40 ± 2.30
	83^w^(72.17)	17.02 ± 8.54

^m^man; ^w^woman.

#### Network analysis of fatigue-related symptom clusters in HIV/AIDS patients

**Figure 2:**
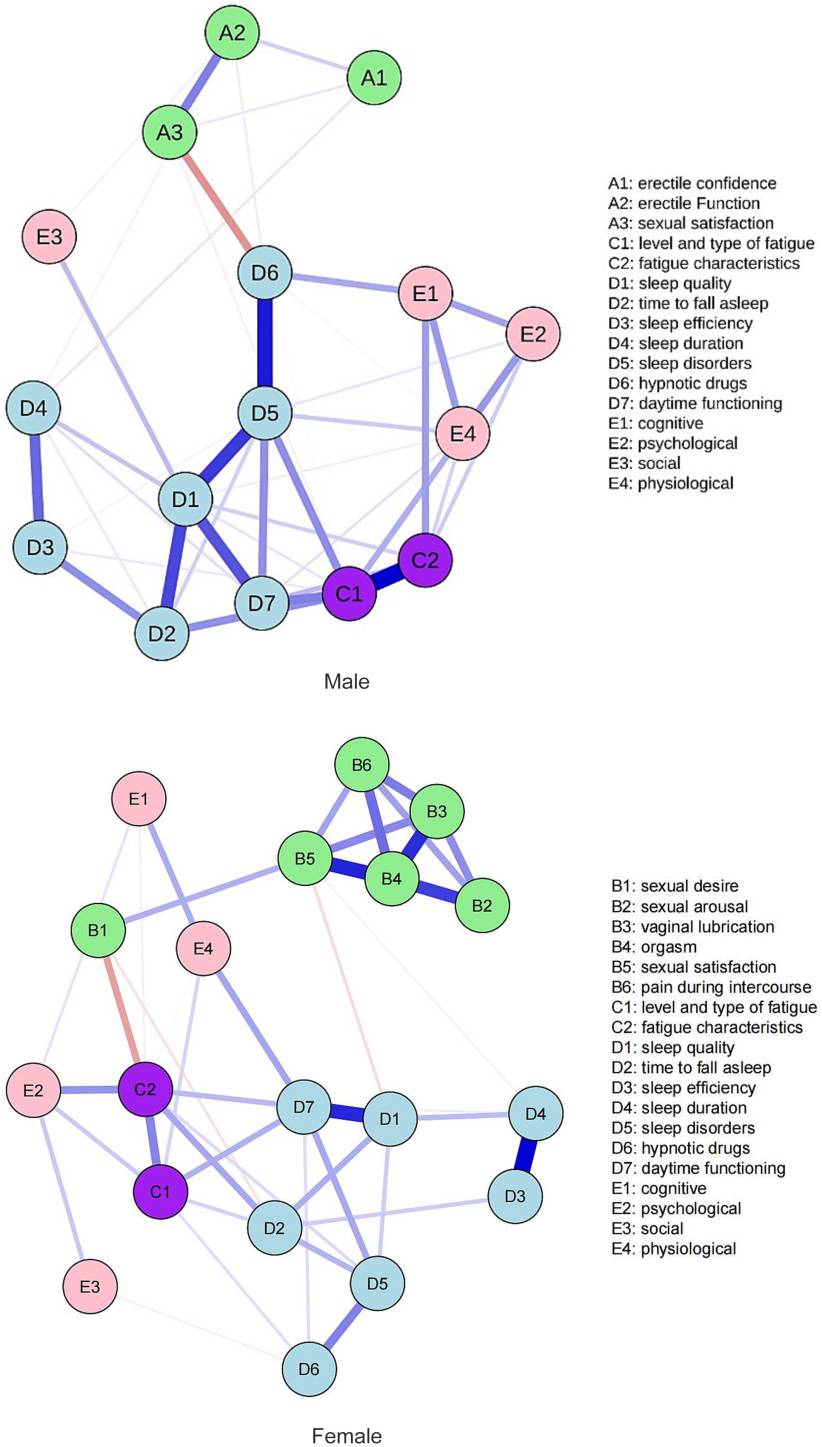
Symptom network of the fatigue-related symptom cluster in patients with HIV/AIDS.

**Figure 3:**
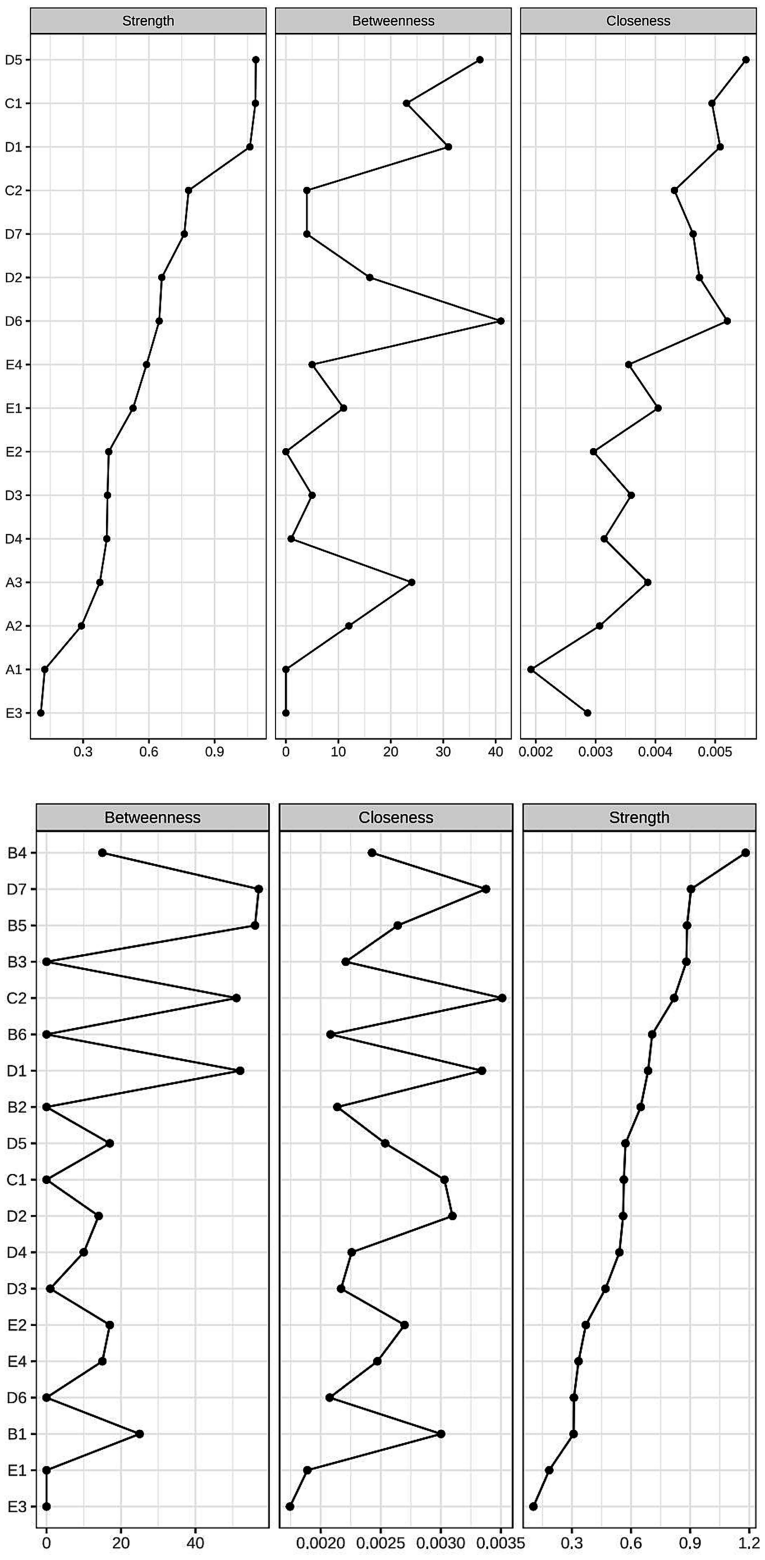
Centrality measures of symptoms within the fatigue-related symptom network in individuals living with HIV/AIDS.

The network centrality indicators reveal that the top three symptoms in terms of strength in the male network are sleep disturbance (D5, rs = 1.61), fatigue severity and type (C1, rs = 1.60), and sleep quality (D1, rs = 1.52). The top three symptoms in terms of closeness centrality are sleep disturbance (D5, rc = 1.52), use of hypnotic drugs (D6, rc = 1.22), and sleep quality (D1, rc = 1.10). The top three symptoms in terms of betweenness centrality are use of hypnotic drugs (D6, rb = 2.00), sleep disturbance (D5, rb = 1.712), and sleep quality (D1, rb = 1.28). In the female network, the top three symptoms in terms of strength are sexual climax (B4, rs = 2.16), daytime function (D7, rs = 1.66), and sexual satisfaction (B5, rs = 1.09). The top three symptoms in terms of closeness centrality are fatigue characteristics (C2, rc = 1.79), daytime function (D7, rc = 1.53), and sleep quality (D1, rc = 1.47). The top three symptoms in terms of betweenness centrality are daytime function (D7, rb = 1.89), sexual satisfaction (B5, rb = 1.84), and sleep quality (D1, rb = 1.65). The symptom network of the fatigue-related symptom cluster in patients with HIV/AIDS is shown in Figure 2, the centrality measures of symptoms within the fatigue-related symptom network in individuals living with HIV/AIDS is shown in Figure 3. Studies have indicated that betweenness and closeness centrality measures tend to be less stable and may not be optimal for assessing node centrality in network analysis (14, 29, 30). Therefore, this study focuses solely on node strength as the criterion for identifying core symptoms. The network analysis results show that the node strength stability coefficient for the male network is 0.60, indicating good stability, while for the female network, the stability coefficient is 0.37, which is considered acceptable. In summary, the core symptoms for male patients are sleep disturbance, fatigue severity and type, and sleep quality. For female patients, the core symptoms are sexual climax, daytime function, and sexual satisfaction.

#### Univariate analysis of factors affecting sleep problems in HIV/AIDS patients

Univariate analysis of factors, including HIV status of sexual partners, duration of ART treatment, income, employment status, and number of sexual partners, revealed statistically significant differences in PSQI scores among HIV/AIDS patients (*p* < 0.05). Detailed results are shown in Table 5.

**Table 5:** Univariate analysis of factors associated with sleep problems among patients with HIV/AIDS (*n* = 422).

Variable	Frequency	PSQI	*t*(*F*)	*P*
Age			1.73^F^	0.18
18–29	65	5.01 ± 3.27		
30–49	229	5.71 ± 3.11		
≥50	128	5.88 ± 3.08		
Gender			0.01^t^	0.99
Male	307	5.66 ± 3.09		
Female	115	5.65 ± 3.28		
Ethnicity			−0.30^t^	
Han	383	5.67 ± 3.20		
Minorities	39	5.54 ± 2.44		
Comorbidities			1.54^t^	0.12
Yes	28	6.54 ± 3.90		
No	394	5.59 ± 3.07		
Partner’s HIV status			−2.20^t^	0.03
Yes	97	5.04 ± 2.82		
No	325	5.83 ± 3.20		
Education level			1.63^F^	0.20
No formal education	9	5.56 ± 2.60		
Lower secondary education or below	240	5.89 ± 3.24		
Lower secondary education or above	173	5.33 ± 3.00		
Marital status			0.53^F^	0.59
Single	209	5.57 ± 3.08		
Married	164	5.63 ± 3.20		
Other	49	6.08 ± 3.16		
BMI			0.51^F^	0.67
Underweight	46	6.09 ± 3.38		
Normal weight	265	5.66 ± 3.11		
Overweight	94	5.39 ± 3.03		
Obesity	17	5.76 ± 3.51		
CD4 + T-cell count			1.61^F^	0.19
<50	12	7.00 ± 4.29		
50–350	106	5.88 ± 3.30		
350–500	117	5.80 ± 2.99		
≥500	187	5.35 ± 3.04		
Living arrangement			0.96^F^	0.38
Owner-occupied housing	176	5.77 ± 3.07		
Rented housing	202	5.68 ± 3.27		
Other	44	5.05 ± 2.68		
Years since HIV Infection			1.97^F^	0.12
<1 year	47	5.12 ± 2.53		
1–5 years	183	5.61 ± 3.22		
5–10 years	85	5.34 ± 2.90		
≥10 years	107	6.22 ± 3.35		
Route of transmission			1.47^F^	0.21
Heterosexual intercourse	234	5.71 ± 3.08		
Homosexual intercourse	143	5.43 ± 3.10		
Intravenous drug use	25	6.00 ± 3.24		
Blood transfusion	2	10.50 ± 6.36		
Unknown	18	5.72 ± 3.49		
Employment Status			7.74^F^	*p* < 0.001
Unemployed	81			
Temporary or short-term employment	69	6.53 ± 3.50		
Permanent or long-term stable employment	239	6.14 ± 3.08		
Agricultural employment	14	4.99 ± 2.78		
Retired	19	7.57 ± 3.46		
Duration of ART			2.86^F^	0.04
<1 year	47	4.49 ± 2.23		
1–3 years	102	5.57 ± 2.94		
3–5 years	95	6.05 ± 3.51		
≥5 years	178	5.80 ± 3.18		
Monthly income (RMB)			5.11^F^	0.002
<1,000	86	6.51 ± 3.63		
1,000–3,000	70	6.17 ± 3.30		
3,000–5,000	144	5.49 ± 3.04		
≥5,000	122	4.95 ± 2.56		
Number of sexual partners				
0	33	5.61 ± 3.11		
1	309	6.26 ± 3.23		
≥2	80	4.61 ± 2.90		
Health insurance coverage			0.89^F^	0.45
Local health insurance	281	5.60 ± 3.14		
No-local health insurance	100	5.66 ± 3.24		
Otherwise	9	4.67 ± 1.58		
Sexual orientation			0.28^F^	0.76
Heterosexual	271	5.70 ± 3.09		
Homosexual	117	5.49 ± 3.31		
Bisexual	34	5.88 ± 2.97		

ART, Antiretroviral Therapy; BMI, Body Mass Index; ^t^CD4 + T-cell count; ^F^One-way Analysis of Variance.

#### Multivariate linear regression analysis of factors affecting sleep problems in HIV/AIDS patients

Multivariate linear regression analysis identified the HIV status of sexual partners, duration of ART treatment, number of sexual partners, and employment status as independent factors influencing sleep problems in HIV/AIDS patients (*p* < 0.05). Detailed results are presented in Table 6.

**Table 6:** Multivariate linear regression of determinants of sleep disturbances in individuals living with HIV/AIDS.

Variable	*B*	Beta	*t*	*P*
(Constant)	4.49		6.26	*P <* 0.001
Partner’s HIV status^1^				
Yes	−0.93	−0.13	−2.66	0.008
Duration of ART^2^				
1–3	1.09	0.15	2.05	0.04
3–5	1.77	0.24	3.29	0.001
≥5	1.27	0.50	2.55	0.01
Number of sexual partners^3^				
1	1.25	0.18	2.24	0.02
≥2	1.92	0.24	3.04	0.003
Monthly income (RMB)^4^				
1,000–3,000	−0.10	−0.01	−0.18	0.85
3,000–5,000	−0.03	−0.01	−0.09	0.93
≥5,000	−0.32	−0.05	−0.52	0.60
Employment status^5^				
Temporary or short-term employment	−0.72	−0.09	−1.17	0.24
Permanent or long-term stable employment	−1.61	0.26	−2.86	0.004
Agricultural employment	0.98	0.89	1.11	0.27
Retired	0.46	0.03	0.59	0.55

*F* = 4.49; *P <* 0.001; ^1^No, ^2^<1 year, ^3^0, ^4^<1,000 RMB, ^5^Unemployed group were used as the reference category.

## Discussion

### Fatigue is the Most common and disturbing symptom reported by HIV/AIDS patients

Fatigue is the most frequently reported and bothersome symptom in HIV/AIDS patients, with the highest prevalence in the fatigue-related symptom cluster. This finding aligns with previous studies (11). HIV-related fatigue (HRF) refers to a subjective experience of tiredness or exhaustion caused by HIV infection, characterized by both physical and psychological fatigue (31). Although HRF does not directly reduce life expectancy, it significantly impacts quality of life, mental health, and treatment adherence, ultimately diminishing treatment outcomes and increasing mortality rates (32, 33). Network analysis results indicate that the node strength for fatigue type and severity (rs = 1.60) ranks second in the male network, suggesting that fatigue holds a central position within the fatigue-related symptom cluster for male HIV/AIDS patients. This emphasizes the need for healthcare professionals to regularly assess symptom changes during follow-up visits, encourage patients to report symptoms proactively, and prevent the onset of HRF. Previous studies have highlighted that unhealthy sleep habits and the misuse of sedative medications can disrupt the sleep patterns of HIV/AIDS patients, leading to poor sleep quality and, consequently, fatigue (34). Therefore, symptom management for fatigue in HIV/AIDS patients should also prioritize addressing sleep issues.

### Sleep problems as a CORE symptom in the fatigue-related symptom cluster of HIV/AIDS patients

The prevalence of sleep problems in male HIV/AIDS patients is 43.32%, while in females, it is 45.22%, which is consistent with previous studies (35, 36). Network analysis results indicate that sleep issues are a core symptom within the fatigue-related symptom cluster in HIV/AIDS patients. Sleep problems can occur at any stage of ART and are one of the most prevalent core symptoms. This could be related to the type of antiretroviral medications used, such as efavirenz, a commonly prescribed non-nucleoside reverse transcriptase inhibitor in developing countries. Efavirenz’s main side effects include neuropsychiatric symptoms, and studies show that 68.1% of HIV/AIDS patients using efavirenz experience sleep disturbances (37). Therefore, it is recommended that healthcare providers identify patients with sleep problems early during follow-up visits. For patients suspected of experiencing sleep disturbances due to efavirenz, timely medication adjustments should be considered. Due to gender differences, when managing sleep problems in patients, male patients should be carefully monitored for symptoms of sleep disturbances, while female patients should receive particular attention for improvements in daytime functioning.

### Higher prevalence of sexual dysfunction in female HIV/AIDS patients compared to males

The prevalence of sexual dysfunction in female HIV/AIDS patients is 72.17%, significantly higher than in male patients (35.83%), which aligns with previous research findings (38, 39). This may be attributed to the fact that women are more likely to experience psychological stress due to the disease. The stigma associated with HIV makes female patients more vulnerable to social discrimination and human rights violations, which can intensify their psychological burden, leading to greater depression and, ultimately, sexual dysfunction (39). Additionally, with increasing age, women’s estrogen levels gradually decline, negatively affecting sexual desire and arousal. The relaxation of the pelvic floor muscles and the sexual atrophy of reproductive organs further decrease their responsiveness to sexual stimuli, thereby increasing the likelihood of sexual dysfunction (40). Studies have shown that hormonal imbalances during menopause, particularly the reduction in estrogen and testosterone levels, are significantly associated with various aspects of sexual dysfunction (38). As ART becomes more widely accessible, the life expectancy of HIV/AIDS patients has increased, and more female patients are entering menopause. However, there is a lack of knowledge regarding the management of menopausal symptoms among these women (41). Therefore, healthcare professionals should provide specialized identification and support for sexual issues in menopausal women with HIV/AIDS.

### The influence of partner’s HIV status on HIV/AIDS patients’ core fatigue-related symptoms

#### The influence of a partner’s HIV infection status on HIV/AIDS patients

The infection status of a sexual partner is an independent factor influencing sleep problems in HIV/AIDS patients, which contrasts with findings from previous studies. In past research (35, 42), the infection status of sexual partners was not specifically addressed, which may account for the discrepancy in results. When both the patient and their sexual partner are HIV-positive, they are more likely to disclose their status to each other in order to gain emotional support, which can reduce anxiety and depression symptoms (43), improve treatment adherence, and indirectly have a positive effect on the patient’s sleep quality. However, when the sexual partner is HIV-negative, disclosure may lead to negative consequences due to the stigma associated with the disease, such as loss of economic support, blame, abandonment, physical and emotional abuse, discrimination, and family breakdown. As a result, most patients choose to conceal their HIV status from their partner (44). In daily interactions, the fear of being abandoned by their partner or transmitting HIV to a non-infected partner creates significant psychological stress, which in turn affects their sleep quality (45). Therefore, healthcare providers should adopt measures to reduce the stigma surrounding the disease for these patients and encourage them to disclose their HIV status to their sexual partners.

#### The duration of antiretroviral therapy

The duration of ART is an independent factor influencing sleep problems in HIV/AIDS patients, which is consistent with previous studies (35). The longer the duration of ART, the higher the likelihood that the patient will experience sleep problems. Research has shown that HIV/AIDS patients who have been on ART for 5–9 years and more than 10 years are 5.65 times and 17.85 times more likely to experience sleep problems, respectively, compared to those who have been on ART for less than 1 year (46). Long-term use of antiretroviral drugs can affect the nervous and endocrine systems, and some medications may induce anxiety, depression, or cause discomfort such as nausea and headaches, all of which can disrupt sleep. Additionally, prolonged ART use may lead to significant impacts on the patient’s daily life, including drug resistance. When resistance occurs, patients need to switch their treatment regimen, which can trigger concerns about disease progression and poor treatment outcomes. This ongoing psychological burden can cause mental stress, making it difficult for patients to relax and fall asleep, thereby reducing sleep quality. In contrast to previous studies (35), this research found that a duration of ART treatment between 3 and 5 years had a more pronounced impact on sleep quality in HIV/AIDS patients. The reasons for this difference remain unclear, and further investigation is needed.

#### Number of sexual partners

The number of sexual partners has been identified as a factor influencing sleep quality among individuals living with HIV/AIDS. However, this finding contrasts with some international studies. For instance, Mazonson et al. (47) found that among older adults living with HIV in the United States, individuals with a single sexual partner reported significantly lower levels of loneliness and depression compared to those with multiple partners. Notably, those with five or more partners exhibited higher levels of loneliness and depressive symptoms, which are known to adversely affect sleep quality (48). The discrepancy between these findings and those from other cultural contexts may be attributed to differing societal attitudes toward sexuality. In Western cultures, more liberal views on sexual relationships may mitigate the psychological impact of having multiple partners. Conversely, in cultures influenced by traditional values, such as those prevalent in China, conservative attitudes toward sexuality may lead to increased psychological distress for individuals with multiple sexual partners, potentially affecting their sleep quality. This cultural conservatism can result in heightened feelings of guilt or shame, which are associated with poor sleep outcomes. Given the limited research on the relationship between the number of sexual partners and sleep quality among HIV/AIDS patients in various cultural settings, further studies are warranted to explore this association comprehensively.

#### Employment status

Employment status has been identified as a significant factor influencing sleep quality among PLWHA, aligning with findings from previous research (49). Several mechanisms may explain this association. Economically, individuals with stable employment typically experience less financial strain, which can reduce stress levels and promote better sleep. Conversely, unemployment can lead to financial hardship, potentially resulting in maladaptive coping behaviors such as smoking, excessive alcohol consumption, and overeating. These behaviors are known to negatively impact sleep quality. Psychologically, unemployment is often linked to increased levels of anxiety and depression. These mental health challenges are closely associated with sleep disturbances (50). Given these considerations, healthcare providers should pay particular attention to the psychological wellbeing of unemployed PLWHA during follow-up visits. Interventions may include counseling, stress management strategies, and referrals to mental health services to address underlying issues contributing to poor sleep quality.

### Limitations

This study has several limitations: (1) The participants were recruited from a single hospital, which may limit the generalizability of the findings. Future research should consider employing a multicenter design to enhance the external validity of the results. (2) As a cross-sectional study, it cannot establish causal relationships or assess the stability of core symptoms over time. Longitudinal studies are needed to examine the temporal dynamics and persistence of these symptoms. (3) The relatively small sample size of female participants may have affected the stability of the network structure analyses for this subgroup. Increasing the number of female participants in future studies would allow for more robust and reliable gender-specific analyses.

## Conclusion

This study employed network analysis to construct a symptom network of fatigue-related symptom clusters among individuals living with HIV/AIDS, identifying sleep disturbances as the central symptom within this cluster. Subsequent multiple linear regression analysis revealed that factors such as the HIV status of sexual partners, duration of ART, number of sexual partners, and employment status are independent predictors of sleep disturbances in this population. These findings underscore the importance for healthcare providers to proactively identify patients experiencing sleep disturbances during follow-up visits. Implementing targeted management strategies may prevent the onset of fatigue-related symptom clusters. Moreover, when patients present with such clusters, focused interventions addressing sleep disturbances can not only alleviate the severity of sleep-related symptoms but also mitigate the intensity of other associated symptoms. This approach can enhance the precision and efficacy of symptom management in individuals living with HIV/AIDS.

## Data Availability

The raw data supporting the conclusions of this article will be made available by the authors, without undue reservation.
